# A Comprehensive Study on the Development of an Isotonic Sports Drink Preserved by UV-C Light Assisted by Mild Heat and Loaded with Yerba Mate (*Ilex paraguariensis* St. Hill.) Extract

**DOI:** 10.3390/foods14091494

**Published:** 2025-04-24

**Authors:** María Luz Kozono, Magdalena Durán Cassiet, Antonella Andreone, Marcela L. Schenk, Sandra N. Guerrero

**Affiliations:** Institute of Food Technology and Chemical Processes (ITAPROQ), CONICET-University of Buenos Aires, Faculty of Exact and Natural Sciences (FCEyN-UBA), Department of Industry, Ciudad Universitaria, Avenida Intendente Güiraldes 2160, Buenos Aires C1428EGA, Argentina; kozono.mluz@gmail.com (M.L.K.); lic.durancassiet@gmail.com (M.D.C.); antonellaandreone.aa@gmail.com (A.A.); marcelaschenk@gmail.com (M.L.S.)

**Keywords:** sports nutrition, functional products, *Ilex paraguariensis*, bioactive compounds, green technology

## Abstract

A unique isotonic sports drink (ISD) was created in this study using a pilot-scale short-wave ultraviolet light (UV-C, 892 mJ/cm^2^, 20 °C) assisted by mild heat (UV-C/H, 534 mJ/cm^2^, 50 °C), which was followed by the addition of yerba mate (*Ilex paraguariensis*) extract (YME). Consumer perception and microbiological and physicochemical stability during refrigerated storage (4 °C/23 d) were assessed for the ISD. *Alicyclobacillus acidoterrestris* (AA) spores and cocktails of yeasts and *Escherichia coli* were considerably inactivated by UV-C/H. The TEM and SEM micrographs showed that AA evidenced sustained severe structural damage. Furthermore, UV-C/H completely inactivated the native microbiota, while YME incorporation increased the initial aerobic mesophilic and mold-and-yeast populations by 0.48 and 0.70 log cycles, remaining stable during storage. YME addition enhanced total polyphenols, flavonoids and antioxidant activity by 2.7–3.7, 16.5–16.7 and 6.6–24.0 times compared to the untreated ISD and ISD-UVC/H samples. Except for sedimentation and turbidity, the other physical characteristics mostly did not change (going from 1494 ± 382 to 2151 ± 106 NTU). The initial 5-HMF value in the ISD was raised by UV-C/H treatment and YME addition. Notwithstanding, it stayed below the allowed threshold throughout storage. ISD-UVC/H+YME had a high overall acceptability score; 60% of panelists gave the drink a score of seven or higher. Additionally, the herbal taste of YME was well liked, and its bitterness was perceived as mild. These findings suggest that including YME and the UV-C/H treatment can produce an ISD of superior quality with distinct sensory attributes.

## 1. Introduction

Recent trends in product launches highlight the significance and advantages of proper hydration, the growing fascination with “superstar” ingredients and their potential health benefits and the importance of maintaining an active lifestyle [1]. The field of sports drink development has undergone substantial changes, propelled by an increasing consumer desire for beverages that offer more than just hydration. A growing market for products combining rehydration properties with additional health advantages exists. Isotonic sports drinks, designed to replace the water, electrolytes and carbohydrates lost during physical activity, are an essential component in the diet of many athletes and active people. They have an iso-osmotic concentration comparable to body fluids (275–300 mOsm/kg of water) for rapid rehydration and avoidance of gastric discomfort [2]. Innovation in this field has led to the search for natural ingredients to improve these drinks’ nutritional profile and organoleptic properties. Nevertheless, achieving a proper formulation is not easy, mainly because multiple ingredients can interact or affect beverage osmolality differently.

The growing consumer interest in innovative products has forced the industry and academia to develop alternative preservation treatments to thermal pasteurization. Traditional thermal treatments ensure safe products and extend their shelf life. Nevertheless, the high temperatures employed can negatively impact these products’ flavor, taste and nutritional value by causing the loss of thermolabile compounds. Furthermore, certain micro-organisms can endure in heat-treated isotonic drinks, including *Alicyclobacillus acidoterrestris*, a spore-producing bacterium that thrives in acidic environments. Its contamination does not cause visible changes in the beverage. However, it significantly alters the flavor and odor, producing unpleasant-smelling “smoky”, “antiseptic” or “disinfectant” flavors due to guaiacol production. These issues may be frequent and represent significant economic losses for juice manufacturing companies [3]. Other primary spoiling agents in fruit juices are fermentative yeasts owing to their physiology and low-pH resistance. Their spoilage mechanism is the generation of significant amounts of ethanol and CO_2_ [4]. Lastly, contamination of fruit beverages with pathogenic micro-organisms, such as *Escherichia coli* O157:H7, also represents an imperative concern. *E. coli* ATCC 25,922 can be a surrogate for *E. coli* O157:H7 [5].

Short-wave ultraviolet light (UV-C, 254 nm) is a promising food preservation alternative among the non-thermal innovative technologies. Its primary inactivation mechanism is based on altering microbial cell DNA through a series of photochemical reactions that impede cell replication. In addition, particular photophysical and photothermal damages have been reported [6]. In addition to producing microbiologically safe products, this technology, considered “green”, has been demonstrated to preserve their nutritional and organoleptic quality well. Some other advantages associated with UV-C light include less energy and lower installation, operation and maintenance costs than other emerging technologies, as well as less demanding space requirements with the possibility of in-line installation [7].

However, this light-based technology significantly decreases its disinfection effectiveness with particles in suspension, compounds that absorb UV-C light or any matrix interference that hinders radiation penetration to the micro-organism [8]. Background microbiota can also limit UV-C light passage, thus impeding overall inactivation [9]. Therefore, it is recommended to use microbial cocktails in microbial challenge tests, as they have proved to be more resilient to the UV-C treatments than single inoculum. Moreover, applying UV-C light under a hurdle approach, combined with other stressors, could enhance its effectiveness [6]. The literature has vast information on combining UV-C light and mild heat to obtain safe products with good nutritional and organoleptic quality [10,11,12,13]. Finally, studying pilot-scale processing is crucial, as it bridges the gap between laboratory-scale experiments and industrial applications, ensuring scalability and practical feasibility.

Among the latest innovations in functional beverages that include herbal ingredients, yerba mate (*Ilex paraguariensis*, St Hill) is a plant native to South America and a popular infusion in this region. It is consumed in its industrialized form, denominated as aged canchada. This term refers to the final industrialization step before commercialization, which involves blanching, two drying steps, grinding and storage [14]. During storage, the yerba mate leaves acquire adequate organoleptic characteristics (aroma, flavor and color). However, for some decades now, yerba mate has gained global attention due to the extensive scientific evidence reported in the literature regarding its multiple health benefits, including antioxidant, anti-inflammatory, anti-cancer and anti-obesity properties. These attributes have been linked to the high content of relevant bioactive compounds like polyphenols, xanthin alkaloids and flavonoids [15]. Among the xanthin alkaloids, significant amounts of caffeine can be found in yerba mate, which promotes alertness, excitement, energy and an elevated mood [16]. The inclusion of yerba mate in a sports nutrition product could not only improve its nutritional value but also incorporate bioactive compounds that enhance recovery and physical performance, in addition to an exciting combination of aromas and flavors of herbs and fruit that could be attractive for those consumers interested in distinctive flavors. Moreover, to date, caffeine used as a food additive has primarily come from synthetic sources. Although it has the same effects as caffeine from natural sources, the issue is that hazardous solvents, such as dimethyl sulphate, are used in its production, raising concerns about safety and the environmental impact [17].

Several products containing yerba mate extracts (YME) have been developed, for instance, orange-tangerine juice [18], corn-based snacks [19], carrot pickles [20], fresh cheese [21], sports gel [22]. However, there is still no scientific evidence regarding the development of isotonic sports drinks loaded with yerba mate as a source of polyphenols and caffeine.

This study aimed to develop an isotonic sports drink treated at a pilot scale with UV-C light, assisted or not by mild heat (50 °C) and loaded with yerba mate extract as a source of bioactive compounds. Furthermore, the product’s physicochemical and microbial stability throughout storage and the consumer response on sensory and emotional levels were studied and statistically analyzed.

## 2. Materials and Methods

### 2.1. Materials and Chemicals

A local producer (ECA Agroindustria, Concordia, Entre Ríos, Argentina) kindly supplied the frozen and concentrated orange juice (65 °Brix) without any preservatives used in this study.

The aged-canchada yerba mate (YM) leaves were generously donated by Cooperativa Agrícola (Colonia Liebig Ltda, Corrientes, Argentina). The aged-canchada industrialization stage was selected, since the highest content of phenolic compounds and total antioxidant activity can be achieved at this stage in the yerba mate leaves [23]. Food-grade sucrose was used. Sodium chloride and potassium dihydrogen phosphate, as the electrolytes of the isotonic beverage, were purchased from Biopack^®^ (Buenos Aires, Argentina).

### 2.2. Isotonic Sports Drink Design

In order to achieve a proper formula for the orange isotonic sports drink (ISD), three main factors were taken into account: an averaged formula from the literature [24,25], the osmolality of every ingredient and that corresponding to the final product and the chemical composition of the industrial juice, as provided by the manufacturers. The optimal ISD recipe was developed to replicate commercially available sports drinks’ sugar, electrolyte and energy levels. It included 20% *v*/*v* reconstituted commercial orange juice (4.3 °Brix), 2.3% *w*/*v* sucrose, 0.04% *w*/*v* NaCl and 0.4% *w*/*v* YME mixed with tap water.

#### Osmolality Study

Osmolalities were assayed using the freezing point depression method in an osmometer (Osmomat 3000, Gonotec, Berlin, Germany). Different systems were developed and measured considering the reference values of osmolality of various commercial ISDs and the concentration ranges of each component in the formula derived from the literature. Osmolality values were determined for the following ingredients: 0.01–0.32% sodium chloride, 0.006–0.125% potassium dihydrogen phosphate, 3.5–20.0% sucrose, 0.4% yerba mate extract, 1–10% reconstituted commercial orange juice, tap water, filter water, mineral water and designed recovery drinks. The osmolality of the final formula was expressed as mOsm/kg water.

### 2.3. Isotonic Sports Drink Processing

The ISD was processed in a self-made pilot-scale UV-C device described in detail in ref. [22]. The UV-C Dean flow reactor, which operated via liquid passes, consisted of a stainless-steel compartment with twelve UV-C lamps (Phillips T8/36 W; UV-C lamp efficiency: 41.7%) arranged in and around a fluorinated ethylene propylene (FEP) coiled tube (UV-C transparency: 0.98–0.99; diameter: 19 mm; length: 13.9 m). The ISD entered the UV-C chamber through sanitary piping at 380 L/h, reaching a turbulent flow (Reynolds number = 3548–6389), as recommended by the U.S. Food and Drug Administration [26] for UV-C processing. The Dean number was 773–1392, and the ratio between the tube (D) and coil (Dc) diameters was 0.05, ensuring proper system mixing due to secondary vortices.

The delivered UV-C fluences were previously estimated by Fenoglio et al. [18], adapting the method proposed by Rahn [27] to the recirculation mode. This method uses an iodide/iodate chemical actinometer.

Validation studies of single UV-C (delivered fluence _(Actinometric)_ = 892 mJ/cm^2^, 20 ± 1 °C) or UV-C assisted by mild heat (UV-C/H, delivered fluence _(Actinometric)_ = 534 mJ/cm^2^, 50 ± 1 °C) treatments were performed. The selection of UV-C fluences was based on previous microbial challenge studies using *E. coli* ATCC 25,922 as a surrogate for the pathogen strain *E. coli* O157:H7. The aim was to achieve five log reductions, as stipulated by the FDA [28]. To choose the best ISD processing treatment, an experimental validation of two equivalent preservation treatments was performed. Then, sensory studies were assayed to evaluate consumer perception of the treated products.

#### Validation Study

The microbial validation studies were performed using *Alicyclobacillus acidoterrestris* ATCC 49,025 spores (AA) and cocktails of *Escherichia coli* (ECC) and yeasts (YC). The ECC comprises *E. coli* ATCC 8739, *E. coli* ATCC 11229 and *E. coli* ATCC 25922, the last being a suitable surrogate for *E. coli* O157:H7, the pathogenic strain [5]. For YC, *Saccharomyces cerevisiae* KE 162, *Zygosaccharomyces bailii* NRRL 7256, *Pichia anomala* NRRL 3668 and *Candida parapsilosis* ATCC 22,019 were the strains selected as representative spoilage agents in fruit juices. AA is another representative spoilage agent in the spotlight, as it causes fruit juice incidents due to its heat-resistant spores.

The AA inoculum was prepared following the methods stated by Kozono et al. [29] and stored at −18 ± 1 °C until use. We followed the technique detailed in Kozono et al. [22] to prepare the cocktail’s inocula. Before treatment, the ISD was inoculated to achieve a final concentration of approximately 10^5^–10^6^ CFU/mL and immediately processed in the UV-C unit. Samples were analyzed for survivors according to the procedures detailed in ref. [9]. Every assay was performed in duplicate. The results were expressed as Log N/N_0_ (N: CFU/mL after treatment; N_0_: initial number of CFU/mL).

### 2.4. Yerba Mate Extract Development

The method applied by Kozono et al. [22] was followed to obtain the YME. Briefly, 10 g of coarsely ground YM leaves was added to 200 mL of distilled water in a 600 mL double-wall cylindrical vessel. The vessel was connected to a thermostatically controlled water bath (Arcano, Model Chilling MP-100, Madrid, Spain) set at 20 °C. The mixture was sonicated for 7 min using an ultrasound (US) processor (Vibracell VCX750; 750 W; 20 kHz; Sonic Materials Inc., Chicago, IL, USA). A 13 mm titanium probe submerged in the solvent was utilized, and the amplitude was set at 0.11 W/mL. Once the extraction proceeded, the sonicated samples were centrifuged (1475× *g*, 10 min) (DCA-300 RVT R, Presvac, Argentina), and the supernatant was lyophilized (Stokes model 21, Philadelphia, PA, USA).

### 2.5. Yerba Mate Extract Addition to the Isotonic Sports Drink

It is essential to clarify that although the YME was considered in designing the final formulation due to its contribution to the product’s osmolality, it was added to the ISD after the UV-C treatment. This decision was based on the available bibliographic evidence regarding the photoprotector activity of YM, which could hinder decontamination processing [30]. The processed UV-C and UV-C/H sports drink samples were immediately loaded with 0.4 g of YME per 100 mL of beverage.

### 2.6. Sensory Studies

Given that both treatments (UV-C and UV-C/H) were effective, sensory studies were carried out to define the processing to be applied to the ISD. One hundred and seventeen unpaid and untrained consumers (55% male and 45% female) took part in the tests. They were selected from among the students and staff at Buenos Aires University who reported exercising at least once a week, and they ranged in age from 18 to 45 years old. Each panelist evaluated two samples of ISD treated with UV-C or UV-C/H and loaded with 0.4% YME (ISD-UVC+YME and ISD-UVC/H+YME, respectively). The samples were presented to the volunteers, following a random sequential monadic order, in white plastic cups at a usual serving temperature (20 mL, 5–7 °C). The sensory tests were conducted under white light in the sensory laboratory of the FCEyN-UBA, with six individual booths constructed and provided with all the necessary elements established by ISO 8589 [31] as guidance. All the panelists performed the consumer field and the check-all-that-apply tests within the same session.

#### 2.6.1. Consumer Field Test

The questionnaire used in this study was performed in a special session by a group of eight trained members, following the guidelines established by Lawless and Heymann [32]. In the consumer field test, the panelists (117) initially evaluated the overall acceptability of the samples on 9-point hedonic scales. Then, the attributes were measured using 5-point acceptability, intensity or just-about-right (JAR) scales. The evaluated attributes included herbal aroma and herbal taste, as well as orange aroma, orange taste, salty taste, bitterness and sweetness. The herbal taste was measured using liking and adequacy (JAR) scales, while saltiness was evaluated on an intensity scale. Finally, the panelists measured the adequacy (JAR) of the attributes of herbal aroma, orange taste, orange aroma, bitterness and sweetness.

#### 2.6.2. Check-All-That-Apply Test

In the check-all-that-apply (CATA) test, the panelists completed a questionnaire right after their sample tasting. They were asked to select all the sensory and non-sensory (emotions) terms from a checklist that they considered appropriate to describe the tasted sample [33]. The checklist included the following descriptors: artificial taste, fresh/refreshing, sour, persistent taste (aftertaste), natural, orange taste, herbal taste, strange taste, pleasant aroma, pleasant body, healthy, astringent, ideal for consuming while working out, thirst-quenching. This questionnaire was also used to evaluate consumers’ perceptions of an ideal ISD [34]. The CATA counts were added together, and the resulting contingency table was used in the following analysis.

### 2.7. Study of the Morphological Damage Induced by the UV-C/H Treatment

Transmission electron microscopy (TEM) and scanning electron microscopy (SEM) studies were conducted to investigate AA inactivation through a morphological point of view in the developed ISD using the proposed treatment.

For TEM, the samples were washed with phosphate-buffered saline (PBS), centrifuged at 1475× *g* for 5 min, and the resulting supernatant was removed. The pellet was resuspended in a solution of 2% glutaraldehyde for 24 h at 5 °C to preserve the morphology and structure of the spores, rinsed with 0.1 M phosphate buffer and post-fixed with 1% OsO_4_ in 0.1M phosphate buffer for 17 h at 4 °C. After fixation, samples were washed with distilled water, dehydrated with alcohol series and embedded in Durcupan (Sigma-Aldrich, USA). Ultrathin sections stained with lead citrate and uranyl acetate were examined using an Electron Microscope Zeiss EM 109T (Lanais-Mie, UBA-CONICET, Buenos Aires, Argentina). Magnification ranging from 85,000× to 140,000× for sizes around 50–100 nm was applied. Untreated cells were subjected to a similar fixation procedure and served as controls.

For SEM analyses, treated and untreated samples were coated with platinum using a sputter coater for 50 s with a current of 30 mA. The images were observed with a Carl Zeiss NTS- Supra 40 Scanning Electron Microscopy (Moringen, Germany). A 3 kV acceleration voltage was applied, and magnification was set at 50,000× for sizes around 200–300 nm.

### 2.8. ISD-UVC/H+YME Storage Studies

After the preliminary studies and once the ISD processing was selected, a global cold storage study was performed (23 days, 4 ± 1 °C). Immediately after treatment, the system was loaded with 0.4% YME, aseptically dispensed into 15 mL caramel flasks and stored at 4 ± 1 °C. Two control samples were also analyzed to evaluate the effects of individual treatments (UV-C/H processing and the addition of YME). Consequently, the assayed samples were labeled as follows: ISD-C: untreated ISD without YME addition (control 1); ISD-UVC/H: UV-C/H treated ISD without YME addition (control 2); and ISD-UVC/H+YME: UV-C/H treated ISD with YME addition.

At predetermined intervals during storage, three flasks of each condition were taken for the corresponding analysis.

#### 2.8.1. Native Microbiota Study

The evolution of the ISD native microbiota, including yeast and mold, total aerobic mesophilic and coliform populations, was monitored throughout the storage period (23 days, 4 °C), with samples taken at predetermined intervals (24–72 h). Yeasts and molds were cultured on chloramphenicol glucose agar (CGA, five days at 25 ± 1 °C), while total aerobic mesophiles and coliforms were determined on plate count agar (PCA, 37 ± 1 °C for 72 h) and MacConkey agar (MC, 48 h at 37 ± 1 °C), respectively. Plots of log N/N_0_ versus storage time were generated. Analyses were conducted in duplicate.

#### 2.8.2. Physicochemical Characterization

Some physicochemical parameters were studied at 0, 5, 9, 15 and 23 days of storage (4 °C) of the ISD samples described above.

A handheld tristimulus reflectance spectrocolorimeter (Minolta Co. Model CM-508-d, Osaka, Japan) was used to measure the color of the ISD samples. The components of the CIELAB space, L*(lightness), a*(green-red) and b*(blue-yellow), were determined for illuminant C at a 2° standard observer. Three milliliters (3 mL) of the sample was analyzed using a 1.4 measuring aperture with white and black backgrounds, as described by Ferrario et al. [10]. Measurements were taken in triplicate.

The pH, total soluble solids content (TSS, °Brix) and turbidity of the samples were measured using a pH meter (PerpHecT 310 Orion, London, UK), a handheld digital refractometer (Palette PR-101, ATAGO, Tokyo, Japan) and a turbidimeter (LaMotte 2020we, Chestertown, MD, USA), respectively. Three replicates of each measurement were used.

The Folin–Ciocalteu method [35] was used to determine total phenolic content (TPC). A calibration curve of gallic acid (ranging from 0.0025 to 0.125 mg/mL) was prepared, and the following equation was obtained: y = 9.5327x + 0.0173, R^2^ = 0.99. The results were expressed as mg of gallic acid equivalents per milliliter of the sample, GAE/mL. The absorbance was measured at 740 nm using a UV–VIS spectrophotometer (SP-UV1000, Dragon Lab, Carlsbad, CA, USA).

The total antioxidant activity (TAA) was assessed using the DPPH (TAA_DPPH_) and ABTS (TAA_ABTS_) radical scavenging methods, as described in detail by Fenoglio et al. [9]. For the DPPH procedure, the absorbance was measured at 515 nm. In the case of the ABTS method, the absorbance was determined at 734 nm. The calibration curve was also obtained using Trolox as a standard. TAA_ABTS_ values were calculated from the calibration curve (y = 5.6229x − 0.068, R^2^ = 0.99) and expressed as mg of Trolox equivalents per milliliter (mg Trolox Eq/mL).

The flavonoid content (FC) of the ISD-C, ISD-UVC/H and ISD-UVC/H+YME samples was determined using a colorimetric method based on Laouini et al. [36]. The absorbance was measured at 510 nm. The equation corresponding to the calibration curve of catechin (Sigma Aldrich, St. Louis, MO, USA) was as follows: y = 0.8626x − 0.0100, R^2^ =0. 99. FC was expressed as mg of catechin equivalents per milliliter (mg catechin Eq/mL).

5-hydroxymethylfurfural (5-HMF) was quantified as described by Rattanathanalerk et al. [37]. The wavelength used was 443 nm. 5-HMF (99% *w/w*, Sigma Aldrich, MO, USA) calibration curve (y = 0.1024x + 0.0358, R^2^ = 0.99) was used to quantify the HMF concentration in the systems, and the values were expressed as mg of 5-HMF per liter (5-HMF mg/L).

#### 2.8.3. Turbiscan Stability Index

Measurements were conducted at room temperature using Turbiscan^®^ CLASSIC 2 (Formulaction Co., L’Union, France) to evaluate the stability of the suspension. At day 0, Turbiscan glass cells were filled with 6 mL of each sample (approximately 55 mm in height), and backscattering readings were recorded at an angle of 135° from the incident light during storage (23 days, 4 ±1 °C).

We used the measurable change in the backscattering signal along the sample height to quantify the destabilization phenomena in the samples. This process resulted in the Turbiscan^®^ Stability Index (*TSI*), a dimensionless number that can be calculated as Formula (1):

(1) $$TSI\;(t)=\frac{1}{N_{h}}\sum_{t_{max}=1}^{t_{max}}\sum_{{Z_{i}=Z}_{min}}^{Z_{max}}\left|BST\;\left(t_{i};\;z_{i}\right)-\left.BST\;\left(t_{i-1};\;z_{i}\right)\right|\right.$$

where *TSI* is the Turbiscan Stability Index; *t_max_* is the measurement point corresponding to the time *t* at which the *TSI* is calculated; *Z_min_* and *Z_max_* are the lower and upper selected height limits, respectively; *N_h_* is the number of height positions in the selected zone of the scan; BST (*t_i_*; *z_i_*) and *BST* (*t_i_***_−_**_1_; *z_i_***_−_**_1_) are the considered backscattering signal for a given scan “*i*” and the previous one “*i* − 1”, obtained at a given *z*.

*TSI* was used to compare the samples’ stability objectively [38]. The higher the *TSI* values obtained, the lower the sample stability.

### 2.9. Statistical Analysis

One-way analysis of variance (ANOVA) was used to evaluate the significance of the microbial strain and treatment effects (UV-C or UV-C/H) on the inactivation achieved. The significance level was set at *p* < 0.05. In case significant differences were found, Tukey’s HSD test was performed. Homoscedasticity and normality assumptions were verified through analysis of the scatterplot of studentized residuals versus studentized predicted values and the Q–Q plot, respectively. If necessary, outliers were removed from the data set.

Multivariate analysis of variance (MANOVA) was used to determine differences between ISD-C, ISD-UVC/H and ISD-UVC/H+YME, considering physicochemical properties and storage time. In case significant differences were found, the Hotelling test with Bonferroni correction was used. Two-way ANOVA was used to evaluate the influence of UV-C/H treatment, the addition of YME and storage time on the evolution of physicochemical parameters’ properties. Tukey’s HSD test was used to determine significant differences among samples. Homoscedasticity and normality assumptions were verified following the procedure described above. The significance level was set at *p* < 0.05. All statistical analyses were performed using InfoStat 2009 (InfoStat Group; FCA-UNC, Córdoba, Argentina).

One-way ANOVA was also performed to identify significant differences between the ISD processed using the UV-C and UV-C/H treatments, according to the independent factor “treatment” and the scores assigned by the panelists in the consumer field test.

Cochran’s Q test was used to identify the attributes that significantly influenced discrimination among the isotonic sports drinks. The results were considered significantly different at a significance level of 5%. Correspondence analysis (CA), based on Chi-square distances, was applied to the frequency tables of ISD-UVC+YME, ISD-UVC/H+YME and an ideal product to generate a bi-dimensional plot of the samples and the attributes selected in the CATA test. These statistical analyses were performed using XLStat 2020.4.1.

## 3. Results and Discussion

### 3.1. Isotonic Sports Drink Design

An orange isotonic sports drink was developed. The osmolality values were 329 ± 5 mOsm/L for ISD-C (3.77 ± 0.01 pH; 1494 ± 93 NTU; 6.9 ± 0.1 °Brix; absorption coefficient_254_: 0.233% *v*/*v^−^*^1^; 59% UVT) and 351 ± 7 mOsm/L for ISD-UVC/H+YME (3.81 ± 0.01 pH; 2151 ± 106 NTU; 7.1 ± 0.2 °Brix). To obtain these values, we considered the linear relationship between the osmolality and the concentration of sucrose, sodium chloride, potassium dihydrogen phosphate and concentrated juice, which was observed in the tested ranges. Furthermore, considering the significant contribution of the electrolytes, sugar and energy of the concentrated orange juice, the final formula was as follows: 20% *v*/*v* reconstituted orange juice (4.3 °Brix), 2.3% *w*/*v* sucrose, 0.4% *w*/*v* YME and 0.04% *w*/*v* NaCl. It is important to highlight that the UV-C light treatment did not affect beverage osmolality, with the osmolality of ISD-UVC/H being 332 ± 5 mOsm/L.

Although the osmolality of the ISD samples was higher than that of body fluids (280–290 mOsm/L), the value reached was comparable to those corresponding to a commercial isotonic drink available on the market (Gatorade^®^). The carbohydrate content was set at 6% *w*/*v*, a value lower than the concentrations (>8–10% *w*/*v*) that usually inhibit gastric emptying and reduce the available fluid for absorption with the consequent gastrointestinal discomfort [2]. Regarding potassium salts, the concentrated juice contribution was sufficient to reach the desired amount in the ISD; thus, potassium salt addition was not required. It was necessary to add NaCl, reaching a final concentration of 420 mg/L of sodium. According to Brouns and Kovacs [39], this amount is adequate to promote, along with sugars, the absorption and retention of water. It was a compromise between obtaining good rehydration but, at the same time, not causing gastrointestinal discomfort and achieving an acceptable flavor [2].

### 3.2. Selection of the UV-C Treatment

#### 3.2.1. Validation Study

**Figure 1:**
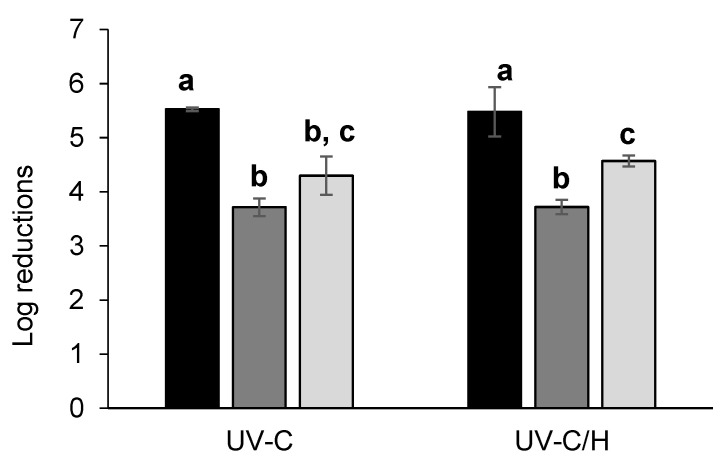
Log reductions in *E. coli* cocktail (■), yeast cocktail (■) and *A. acidoterrestris* spores (■) achieved in isotonic sports drink processed by single UV-C (892 mJ/cm^2^, 20 ± 1 °C) or assisted by mild heat (534 mJ/cm^2^, 50 ± 1 °C; UV-C/H). One-way ANOVA with post hoc Tukey’s HSD test; different letters above the bars indicate statistically significant differences (*p* < 0.05). Standard deviation (I).

The log reductions in ECC, YC and AA achieved in the ISD samples processed using UV-C and UV-C/H are shown in Figure 1. After a single UV-C treatment (892 mJ/cm^2^ at 20 ± 1 °C), ECC, YC and AA were reduced by 5.5, 3.7 and 4.3 log cycles, respectively, demonstrating moderate to high inactivation depending on the considered micro-organism. ECC was the most sensitive cocktail to UV-C light among all tested micro-organisms (*p* < 0.05, Figure 1). Fenoglio et al. [40] observed a similar trend in a challenge test employing a range of micro-organisms in both clear and turbid juices (absorption coefficient at 254 nm: 0.12–0.39 cm^−1^; UVT%: 40.7–75.9%; 166–7667 NTU) processed by UV-C light at laboratory scale (1720 mJ/cm^2^, 20 ± 1 °C). They also reported greater sensitivity of bacteria (*E. coli* strains ATCC 35218, 11229 and 25922, *L. innocua* ATCC 33090 and *P. fluorescens* ATCC 49838) to UV-C light compared to yeasts (*S. cerevisiae* KE 162, *Z. bailii* NRRL 7256 and *C. parapsilosis* ATCC 22019) in all the evaluated matrices. Gomez-Sánchez et al. [41] studied the inactivation of *E. coli* ATCC 25,922 and *S. cerevisiae* (an isolated strain from the Food Microbiology Laboratory at Universidad de las Americas Puebla) in commercial pomegranate juice (3.45 pH; 11.38 °Brix) using UV-C-coiled equipment. They found that *E. coli* exhibited greater sensitivity to UV-C light (6369 mJ/mL, 20.0 ± 0.2 °C) than to yeasts, achieving 4.38 log reduction versus less than 1 log cycle, respectively.

Regarding AA, the results were promising, as the level of inactivation achieved exceeded the typical contamination levels in commercial fruit juice production [42]. Therefore, UV-C light presents a promising alternative for controlling *A. acidoterrestris* spores in industrialized fruit juices. Additionally, the literature has limited information on the inactivation procedures performed at the pilot scale for AA spores, making these results particularly relevant. Other studies reported variable inactivation of *A. acidoterrestris* spores depending on the UV-C dosage, scale and type of reactor. For instance, Sauceda-Gálvez et al. [43] evaluated the efficacy of UV-C on *A. acidoterrestris* CECT 7094 inoculated in clarified apple juice (0.56 ± 0.02 NTU; α_254_ = 7.8 ± 1.9 cm^−1^; 11.1 °Brix; pH: 3.72 ± 0.18) using a thin-film concentric-ring-type UV-C device under recirculation mode. They achieved a lethality greater than 4 log reductions when applying fluences of 10.6, 14.3 or 21.5 J/mL, with UV-C effectiveness unaffected by the temperatures ranging from 20 °C to 60 °C. The highest inactivation (5.8 log_10_) was observed after applying the highest fluence (21.5 J/mL). Moreover, Tremarin et al. [44] demonstrated the efficacy of UV-C treatment on *A. acidoterrestris* CCT 4384 spores in commercial apple juice (11 °Brix; pH = 3.29; UVT% = 27%), achieving around 5 log reductions with a fluence of 403 mJ/cm^2^ in a laboratory-scale UV-C light batch reactor.

With regard to the UV-C/H treatment, similar inactivation levels were obtained (5.5, 3.7 and 4.6 log reductions for ECC, YC and AA, respectively). Gouma et al. [13] also studied the efficacy of combining UV-C light (3920 mJ/mL, annular flow) with moderate heat (60 °C) against a range of micro-organisms in carrot juice (α_254_ = 40.5 cm^−1^). After the combined treatment, the *E. coli* O157:H7 population decreased by 5.3 log cycles. Additionally, the authors evaluated the inactivation of *S. cerevisiae* STCC 1172 and, contrary to expectations, observed a more significant reduction in this yeast than the evaluated bacteria. However, they concluded that the inactivation was mainly due to the thermal effect, as the survival curves for the yeast under heat treatment overlapped with those corresponding to the combined treatment.

In conclusion, in both cases, a proper level of inactivation of the *E. coli* cocktail, which included the surrogate strain for *E. coli* O157:H7, was achieved, higher than the 5 log cycles required by the FDA regulation [28]. Furthermore, the two proposed treatments were equally effective against yeast cocktails and AA spores, making them suitable preservation methods for liquid food, such as isotonic sports drinks. However, the combined treatment, UV-C/H, required significantly less UV-C fluence to achieve similar inactivation than the single UV-C (534 mJ/cm^2^ versus 892 mJ/cm^2^), which is advantageous for industrial applications, as it involves shorter processing time (12 min against 21 min).

#### 3.2.2. Sensory Studies

##### Consumer Field Test

A consumer field test was conducted to determine how the beverages treated with UV-C or UV-C/H and YME addition (ISD-UVC+YME and ISD-UVC/H+YME, respectively) were perceived by the panelists, thereby determining the best processing method for the ISD from a sensory point of view. One-way ANOVA analysis revealed no significant differences in the overall impression of the tested samples (*p* > 0.05), showing average acceptability values of 6.5 ± 1.5 and 6.4 ± 1.4, respectively, on the 9-point hedonic scale, with a normal distribution of scores between 3.0 and 9.0 verified by the analysis of the corresponding Q–Q plot. However, it is essential to highlight that 56–60% of the panelists rated ISD-UVC+YME and ISD-UVC/H+YME more than 7.0 on the hedonic scale, respectively, suggesting a potential segmentation of consumers, with a significant portion of the panel expressing a preference for a type of drink with noticeable herbal nuances conferred by the YME. This group of individuals could be linked to those consumers described in the literature as generally prompted to try bolder flavors and/or to have multisensory food and beverage experiences [18].

**Figure 2:**
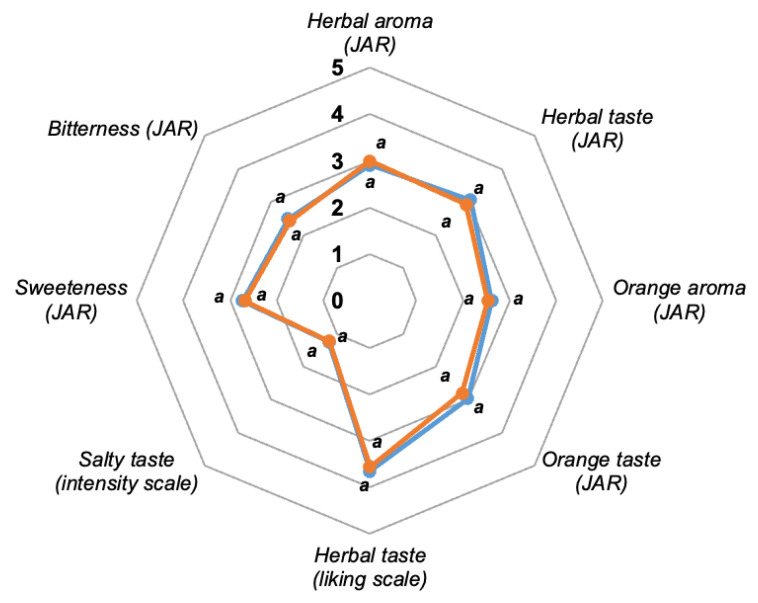
Radar plot for the isotonic sports drink samples treated with UV-C (▬) and UV-C/H (▬) and loaded with 0.4% *w/w* YME, representing the attribute mean values measured on 5-point liking (herbal taste), intensity (saltiness) and JAR (herbal aroma, orange aroma, herbal taste, orange taste, sweetness and bitterness) scales. The average attribute values with the same letter were not significantly different (*p* > 0.05).

Figure 2 depicts the radial plot representing the mean scores for each evaluated attribute. Sensory attributes measured by the just-about-right scale included herbal aroma, herbal taste, orange aroma, orange taste, sweetness and bitterness. For both samples, herbal aroma (2.9–3.0), herbal taste (2.9–3.1), orange aroma (2.5–2.6), orange taste (2.8–3.0) and sweetness (2.70) were close to the “JAR” category. Bitterness averaged the “weak” category (2.4–2.5) but was closer to the adequate value. These results could suggest that the YME did not significantly contribute to the bitterness characteristic of the ISD or that other components of the drink masked it. The ANOVA results showed no significant differences (*p* > 0.05) between the assayed samples. Additionally, the formulation of the ISD was well balanced, as most consumers selected the adequate point on the JAR scale for most attributes. The herbal taste was also measured on a 5-point acceptability scale, considering that, although YM is a popular infusion in Argentina, the sports drink loaded with YME may not appeal to consumers. The panelists expressed that they liked the ISD’s herbal taste (3.7 and 3.6), whether treated with UV-C or UV-C/H, respectively (Figure 2). The herbal taste in the samples was highly acceptable to more than half of the panelists, as evidenced by the percentages of 37.4–39.1% and 15.7–20.0% who gave the drink ratings of 4 (“like slightly”) and 5 (“like a lot”), respectively. The mean scores for salty taste were 1.2 ± 0.5, corresponding to the “without salty taste” category on the 5-point intensity scale (Figure 2). Thus, in addition to their potential health benefits, adding YME to sports drinks could provide natural organoleptic characteristics with unconventional notes, potentially distinguishing this product from those already commercially available.

##### Check-All-That-Apply Test

Cochran’s Q test results indicated that 14 of the 15 terms in the CATA test significantly differentiated the ideal ISD from the ISD-UVC+YME and ISD-UVC/H+YME samples (*p* < 0.05). However, while there were significant differences between the ideal beverage and the UV-C and UV-C/H treated samples, no significant differences were found between the UV-C and UV-C/H samples. The attribute that did not significantly differentiate among the three products was astringent (*p* > 0.05).

**Figure 3:**
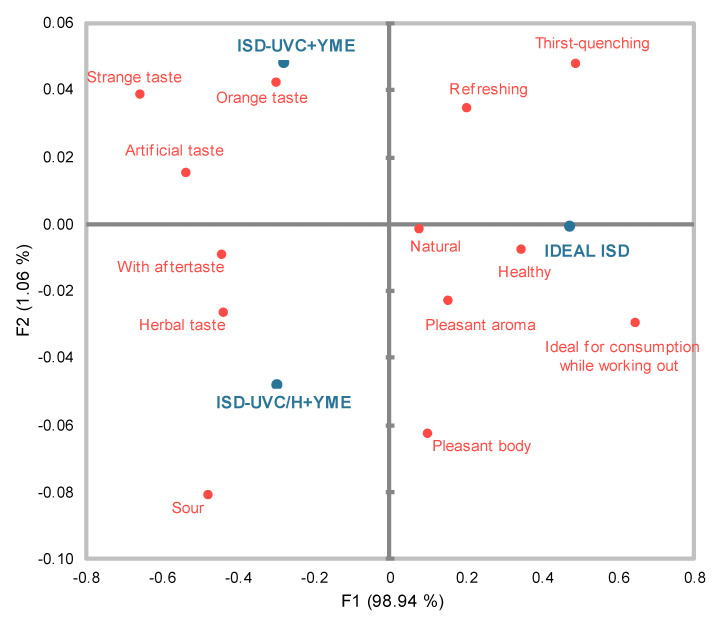
Bi-plot of the correspondence analysis (CA), representing the results generated by consumers via application of the CATA method for the sensory evaluation of isotonic sports drinks treated with UV-C and UV-C/H, both loaded with 0.4% YM extract, as well as the ideal isotonic beverage.

A correspondence analysis (CA) was performed to explore the results from the CATA test and determine the relationships between the terms selected and consumers and the samples, based on the frequency matrix of each term chosen by consumers for each sample. Figure 3 shows the bi-dimensional plot resulting from the CA. The first and second dimensions explain 98.9% and 1.1% of the experimental data variance, respectively.

Although Cochran’s test indicated no significant differences between ISD-UVC+YME and ISD-UVC/H+YME, the panelists detected some terms as more pronounced in one sample than in the other. For instance, the UV-C+YME isotonic drink was associated with artificial, orange and strange tastes. The UV-C/H+YME sample showed negative values on both axes and was described by the panelists as having herbal and sour tastes and with an aftertaste. These findings suggest that applying UV-C light for more than 20 min may impart to the sample a strange taste, while using mild heat (50 °C) to assist a shorter UV-C treatment prevents those changes, making the herbal taste of the added extract more noticeable. Conversely, the ideal ISD was described as natural, with a pleasant body, refreshing, healthy, thirst-quenching and ideal for consumption while working out. It should be noted that the term astringent was omitted from the CA, since no significant differences were found in its frequency of mention after applying Cochran’s Q test.

Even though the proposed ISDs enriched with YME, whether treated with UV-C or UV-C/H, were described as far from an ideal sports drink that consumers expected, several desirable terms associated with an ideal sports beverage—such as refreshing, natural taste and pleasant consistency—were frequently mentioned in their CATA test. Based on the results, further improvements in the formulation could be implemented to more closely approximate the ideal ISD.

### 3.3. UV-C/H Induced Damage

**Figure 4:**
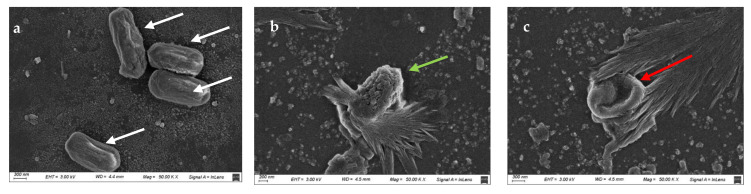
Scanning electron microscopy (SEM) images of (**a**) untreated *A. acidoterrestris* spores, (**b**) and (**c**) *A. acidoterrestris* spores subjected to UV-C light exposure assisted by mild heat (534 mJ/cm^2^, 50 ± 1 °C). The white arrows show untreated spores with a slightly rough surface; the green arrow shows a treated UV-C/H spore with high surface roughness; and the red arrow shows a crushed spore with a central hollow. Magnification was set at 50,000× for sizes around 200–300 nm.

The treatment effect on AA can be observed in SEM and TEM images. Figure 4 corresponds to images obtained by SEM that display the morphological changes in *A. acidoterrestris* spores before and after the UVC/H treatment (534 mJ/cm^2^, 50 ± 1 °C). The untreated spores presented intact membranes with regular contours in their shape. They exhibited a slightly rough surface (Figure 4a, tagged with white arrows). In contrast, AA spores treated with UV-C/H showed a notably increased roughness on their surface (Figure 4b, marked by a green arrow) and a loss of their structural integrity, with a central depression, even hollows (Figure 4c, marked by a red arrow), as a result of damages induced by UV-C radiation.

**Figure 5:**
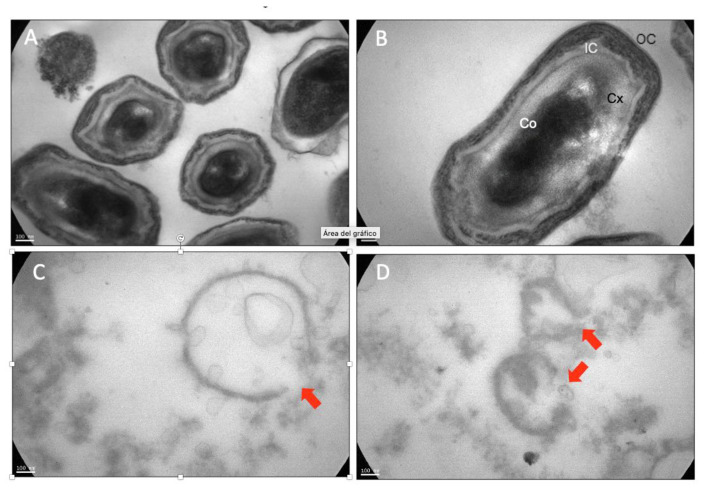
Transmission electron microscopy (TEM) images of (**A**) untreated *A. acidoterrestris* spores, 85,000×; (**B**) magnification of image (**A**), 140,000×; and (**C**,**D**) *A. acidoterrestris* spores subjected to UV-C light exposure assisted by mild heat (534 mJ/cm^2^, 50 ± 1 °C), 85,000×. Magnification was set between 85,000× and 140,000× for sizes around 50–100 nm. Co: core; Cx: cortex; IC: inner coat; OC: outer coat. The red arrows show broken coats.

TEM analyses provided further information on the changes in the inner structure of the spores after the UV-C/H treatment. TEM images showed the untreated spores with intact core, cortex, inner and outer coats preserved (Figure 5A,B). In untreated spores, the different structures were differentiated from each other (Figure 5B). Conversely, in the treated spores, the layers were utterly destroyed, and the coats were broken, giving no possibility of identifying the cortex or the other different structures (Figure 5C,D). Moreover, the spore cores were empty, consistent with the hollowed-cell images obtained by SEM (Figure 4b).

Ferreira et al. [45] evaluated the effect of UV-C radiation (126 mJ/cm^2^) combined with nisin (15.62 μg/mL) on reconstituted orange juice (11 °Brix, pH 4.0) against *A. acidoterrestris* 0244T spores. Consistent with this study, while untreated spores were intact and had a smooth surface, after treatment, the spores changed their morphology, presenting depressions in their shape. Van Luong et al. [46] investigated the inactivation of *A. acidoterrestris* ATCC 49,025 spores in apple juice (11 °Brix, pH 3.8) via high-pressure thermal processing (HPTP) treatments (600 MPa and 80 °C for 5 min). TEM images revealed results similar to ours. While untreated spores presented defined structures, including the core, cortex, inner and outer coats, the membranes ruptured, and intracellular material was lost after treatment.

### 3.4. Storage Stability Studies

Given these findings, the UV-C light treatment assisted by mild temperature (50 °C) was chosen as the ISDs’ preservation method for the subsequent storage stability studies (23 days, 4 °C). This choice aligns better with sustainable practices and industry interests due to the lower fluence required.

#### 3.4.1. Native Microbiota Evolution

**Figure 6:**
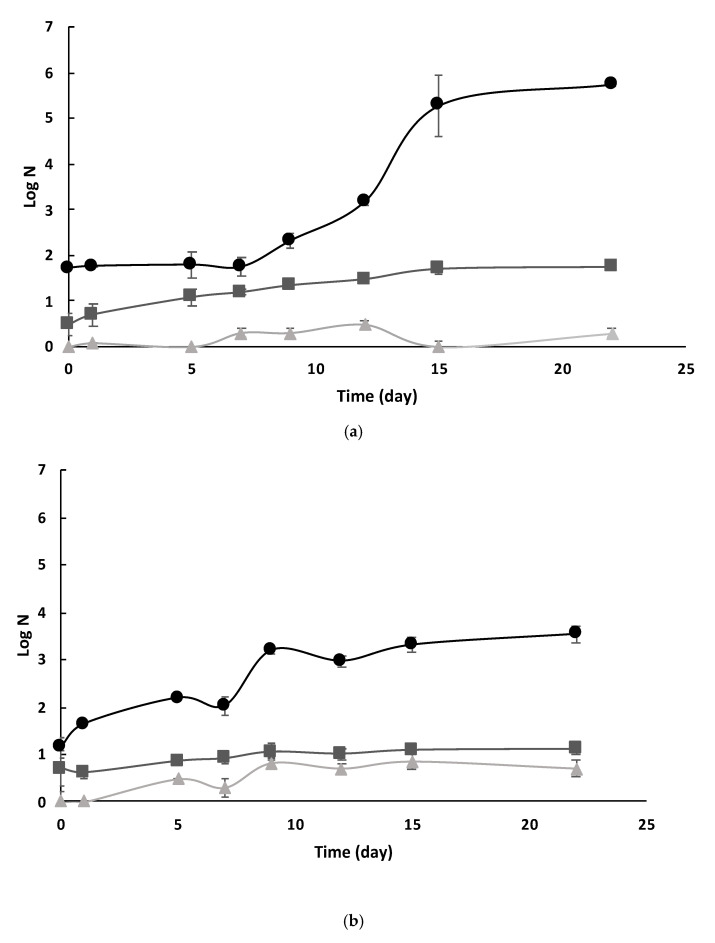
Evolution of native microbiota in ISD-C (●), ISD-UVC/H (▲) and ISD- UVC/H+YME (■) samples during refrigerated storage (4 ± 1 °C). Aerobic mesophiles (**a**), molds and yeasts (**b**). Standard deviation (I).

Figure 6 shows the evolution of the ISD-C, ISD-UVC/H and ISD-UVC/H+YME native microbiota over 23 days of refrigeration storage (4 ± 1 °C). No coliforms were detected in any of the three samples throughout cold storage. The UV-C light treatment assisted by mild temperatures induced a complete inactivation of native microbiota, with a decrease of 1.14 and 1.72 log reductions in yeast and mold and aerobic mesophilic counts, respectively, immediately following treatment in the ISD. In addition, micro-organisms could not recover throughout the storage, unlike the counts in untreated samples, which increased during the studied period (Figure 6a,b).

Regarding the ISD-YME, adding the yerba mate extract to the ISD-UVC/H increased the counts of the aerobic mesophiles by 0.48 log cycles (Figure 6a); in contrast, yeast and molds increased by 0.70 logs (Figure 6b). However, similar to what happened to the ISD-UVC/H samples, the yeast and mold population and the aerobic mesophiles remained constant during the whole refrigerated storage period. La Cava and Sgroppo [11] studied the efficacy of UV-C light (39.6 mJ/mL) assisted by moderate heat (65.0 ± 3.0 °C) in the inactivation of aerobic mesophilic bacteria and yeasts and molds in grapefruit juice (pH 3.12, 9.6°Brix, 2500 NTU, absorption coefficient 42.9 cm^−1^) using a continuous flow reactor. They also assessed the microbial shelf life during refrigerated storage for 28 days and compared the results with untreated samples. In agreement with our study, these authors found that the proposed treatment inactivated all microorganisms, without observing any recovery throughout storage. In contrast, total aerobes and yeast/molds significantly grew during cold storage in the untreated samples. Furthermore, after 21 days of refrigerated storage, the recommended limit for ready-to-eat food (4 log CFU/mL) was surpassed [47].

Regarding the same trend, Santana-Jimenez et al. [48] treated *Agave tequilana* extracts (2.2 °Brix, absorption coefficient 0.48 cm^−1^) at different pH values (4.5, 5.5 and 6.5) with UV-C radiation using a CiderSure 3500 commercial UV-C unit (FPE Inc., Rochester, NY, USA). Similar to our work, they observed a complete inactivation of native microbiota at pH 4.5 when applying a dose of 10.93 mJ/cm^2^, with a reduction in the aerobic mesophilic population, total coliforms and yeast/molds of 2.64, 2.70 and 3.74 log cycles, respectively. Moreover, when working at 33.29 mJ/cm^2^, the native microbiota was completely inactivated across all tested pH values.

#### 3.4.2. Physicochemical Parameters

Some physicochemical parameters were measured immediately after processing and during 23 days of refrigerated storage (4 ± 1 °C) to examine the influence of the UV-C/H treatment and the addition of the YME. Table 1a shows the evolution of pH, TSS, turbidity and color parameters throughout the study period. These parameters remained unchanged after the UV-C/H treatment, with only slight variations during storage. These findings align with previous studies on beverages treated with UV-C light [11,18]. For instance, the pH of ISD-C was 3.77 ± 0.01, with no significant differences observed after UV-C/H processing (3.76 ± 0.01) or the YME enrichment (3.86 ± 0.01). In terms of color, a slight reduction in luminosity was noted after adding the herbal extract. However, this decrease in the L* parameter was insignificant (*p* > 0.05), as can be noted by the general aspect of the samples depicted in Table 1a. Furthermore, according to the two-way ANOVA, the storage time did not significantly affect this parameter (Table 1a).

Turbidity was the parameter that exhibited the most variation, increasing from 1494 ± 382 to 2151 ± 106 NTU, with the addition of YME being the most influential factor (Table 1a). Moreover, Fenoglio et al. [18] developed a functional orange-tangerine juice (12.6 °Brix, 3533 NTU) processed by UV-C light (390 mJ/cm^2^), assisted by mild heat (50 °C) and enriched with a self-made yerba mate extract (0.4 g/100 mL). Their findings align with the results presented in this study, reporting no significant changes in the juice blend’s color parameters, TSS or turbidity after the treatment with UV-C light assisted by moderate temperature. They observed a significant turbidity increase following the addition of the herbal extract. However, while we did not observe significant differences in color parameters after adding the herbal extract (Table 1a), they reported a decrease in a* when the juice was loaded with the yerba mate extract.

Table 1b displays the TPC, FC and TAA values corresponding to ISD-C, ISD-UVC/H and ISD-UVC/H+YME during storage at 4 ± 1 °C. The ISD-UVC/H+YME sample exhibited the highest TPC (1.149 ± 0.092 mg GAE/mL), FC (5.541 ± 0.589 mg catechin Eq/mL) and TAA values (TAA_DPPH_ and TAA_ABTS_: 2.380 ± 0.068 and 8.963 ± 0.016 mg Trolox Eq/mL, respectively) (*p* < 0.05) immediately after processing. In comparison, ISD-C and ISD-UVC/H showed TPC values of 0.308 ± 0.001 and 0.401 ± 0.048 mg GAE/mL; FC values of 0.332 ± 0.014 and 0.335 ± 0.005 mg catechin Eq/mL; TAA_DPPH_ values of 0.099 ± 0.025 and 0.254 ± 0.077 mg Trolox Eq/mL; and TAA_ABTS_ values of 1.348 ± 0.046 and 1.208 ± 0.026 mg Trolox Eq/mL, respectively, at the beginning of storage. No significant differences were observed between both samples. This result indicates that UV-C light treatment combined with moderate heat does not affect the isotonic beverage in terms of TPC, FC and TAA (*p* > 0.05). However, adding yerba mate extract increased the content of these bioactive compounds and their antioxidant activity.

During storage (23 days, 4 °C), the samples evolved with different profiles. The TPC, TAA_DPPH_ and FC values of ISD-C and ISD-UVC/H remained stable throughout the refrigerated storage period (Table 1b). In contrast, ISD-UVC/H+YME exhibited an oscillatory behavior, with a decrease in TAA (TAA_DPPH_: from 2.380 ± 0.068 to 1.493 ± 0.124 mg Trolox Eq/mL; TAA_ABTS_: from 8.963 ± 0.016 to 7.843 ± 0.016 mg Trolox Eq/mL) and FC (from 5.541 ± 0.589 to 4.434 ± 0.007 mg catechin Eq/mL) and an increase in TPC (from 1.149 ± 0.092 to 1.416 ± 0.114 mg GAE/mL) by the end of the storage.

After UV-C light treatment, some physicochemical parameters, such as TPC and TAA, exhibited unpredictable behavior. This behavior could be due to the distinct phenolic compositions in fruits and vegetables, as each phenolic compound reacts differently to UV-C exposure. The variability in antioxidant capacity is also dependent on the phytochemical structure. For instance, Fenoglio et al. [18] observed no significant variations in polyphenol content or in the total antioxidant activity of orange-tangerine juice processed by UV-C light (390 mJ/cm^2^) assisted by mild heat (50 °C). These findings are consistent with the results reported by Türkmen and Takci [49], who found no significant differences in the antioxidant capacity and the content of bioactive compounds of black carrot juice (pH 6.3, 12.0 °Brix) after treatment with UV-C light (150 mJ/cm^2^). Gök [8] studied the effect of UV-C light exposure on freshly squeezed grape juice (pH 4.3, 17.5 °Brix, absorption coefficient 1.00 cm^−1^) and apple juice (pH 3.9, 13.7 °Brix, absorption coefficient 0.32 cm^−1^) using a laboratory-scale UV-C reactor. They also observed that the TPC of grape juice was not significantly altered after the UV-C light treatment (1232 mJ/cm^2^). However, in contrast with our results, they observed increased DPPH radical scavenging activity. Moreover, when the apple juice was exposed to UV-C light radiation (1668 mJ/cm^2^), its total phenolic content and antioxidant activity significantly increased.

Regarding the effect of adding YME, more recently, Neis et al. [20] reported a significant increase in the TPC value of carrot pickles when different concentrations of yerba mate extract (maceration of yerba mate powder < 425 μm, in water (1:10), at 80 °C for 30 min) were added to the pickling brine. Similarly, Rocha Saraiva et al. [21] examined the effect of adding yerba mate extract (1 g of yerba mate leaves with 9 mL of methanol, macerated for 10 min) on the physicochemical characteristics of fresh cheese during storage. After incorporating yerba mate extract into the cheese, they observed a significant enhancement in the concentration of bioactive compounds and total antioxidant activity. Adding 1.0% of YME increased the TPC, TAA_DPPH_ and TAA_ABTS_ 2.3-, 22.9- and 2.7-fold, respectively, compared to the control. However, these parameters were influenced by storage time, showing a reduction in all of them by the end of the storage period.

Although information on enriching isotonic sports drinks with plant-based additives is available, no studies reported using yerba mate extracts. Porfirio et al. [50] elaborated on a natural isotonic sports drink loaded with *Myrciaria jaboticaba* extract (1:2 ratio of peels/pulps to an hydroethanolic solution, macerated for 24 h and dried in a rotary evaporator). In agreement with the present study, the addition of 10% or 12% of the herbal extract enhanced the total phenolic content by 31-fold and 36-fold, respectively.

The 5-HMF content was determined in the treated samples immediately after processing and during refrigerated storage. The isotonic sports drink is rich in sugars, which could be prone to alterations occurring after the UV-C/H treatment and prolonged and inappropriate storage [51], which may lead to the formation of 5-HMF. The metabolite 5-HMF derives from furan, a carcinogenic and cytotoxic compound, and can irritate the mucous membrane of the upper respiratory tract, eyes and skin [52]. According to the Association of the Industry of Juices and Nectars from Fruit and Vegetables for the European Union (AIJN), the established limit for 5-HMF is 20 mg/L [53].

At the beginning of the storage, the ISD-C, ISD-UVC/H and ISD-UVC/H+YME samples displayed values of 0.290 ± 0.096 mg 5-HMF/L, 0.842 ± 0.169 mg 5-HMF/L and 1.428 ± 0.271 mg 5-HMF/L, respectively. This outcome suggests that UV-C radiation induced a slight increase in the metabolite concentration, as did the addition of YME. Regarding the evolution during refrigerated storage, the 5-HMF content oscillated in the three samples analyzed. However, the maximum value recommended by the AIJN was never exceeded [53]. There is available research evaluating the effect of UV-C light on 5-HMF production in different food matrices. Generally, these studies show that the levels of this undesirable byproduct after UV-C exposure are consistently low, which is consistent with our findings. For instance, using a batch benchtop UV-C reactor, Yıkmıs et al. [54] processed *Uruset apple* juice with UV-C light and moderate temperature (97.33 mJ/cm^2^ and 40 °C). The proposed treatment had no significant effect on the amount of 5-HMF. Gök [8] obtained analogous results while examining the effect of a Dean-flow UV-C treatment on the content of 5-HMF in apple (1668 mJ/cm^2^) and grape (1232 mJ/cm^2^) juices. In this study, 5-HMF levels slightly increased after UV-C processing, but in agreement with our results, the recommended value was not exceeded. Conversely, in another study, Santhirasegaram et al. [55] treated *Chokanan mango* juice with high-intensity ultrasound (US—ultrasonic bath, 40 kHz, 68–72 W, 15 or 30 min), followed by UV-C light (352.5 mJ/cm^2^, 15 or 30 min). Only after combining 30 min of US plus 30 min of UV-C exposure, a significant increase in HMF was observed (0.98 ± 0.07 mg/L) compared to the untreated control (0.60 ± 0.03 mg/L).

#### 3.4.3. Particle Sedimentation Study

**Figure 7:**
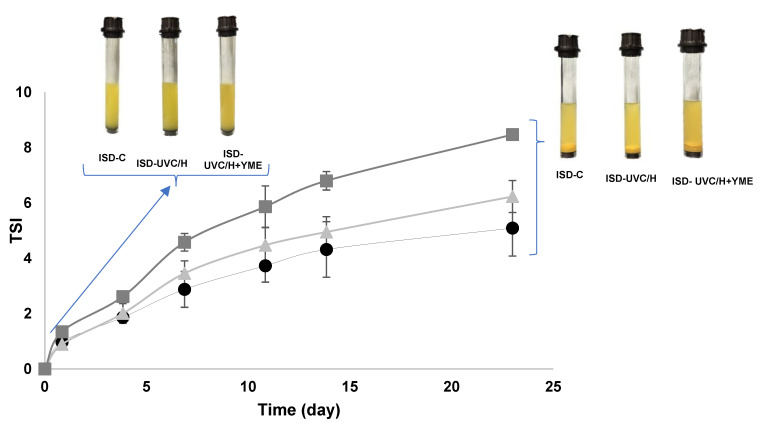
Evolution of the Turbiscan Stability Index (TSI) values in the ISD samples: C (●), UVC/H (▲) and UVC/H+YME (■) over refrigerated storage period (23 days, 4 ± 1 °C). The values are shown as means ± SD of triplicate determinations. Standard deviation (I).

The Turbiscan Stability Index (TSI) is a dimensionless number that allows for a quick and robust quantification and comparison of the evolution of the sample destabilization phenomenon. ISD-C, ISD-UVC/H and ISD-UVC/H+YME were evaluated over 23 days in cold storage to study product destabilization during shelf life. Figure 7 depicts the evolution of TSI values over time. The samples were unstable during storage, with particle migration resulting in sedimentation. ISD-UVC/H+YME was the least stable suspension, exhibiting a higher slope and TSI values. The primary factor leading to sedimentation is particle density. Adding YME increased the density of solids, resulting in a higher gravitational force and, consequently, a faster sedimentation rate. However, this may not be entirely negative, as a precipitated solution can be a typical characteristic of this type of drink.

## 4. Conclusions

In this study, an isotonic sports drink (ISD) was successfully formulated, reaching an osmolality comparable to the commercially available isotonic beverages. The short-wave ultraviolet light used for ISD processing at the pilot scale, as a single stress factor (UV-C, 892 mJ/cm^2^, 20 °C) or combined with mild temperature (UV-C/H, 534 mJ/cm^2^, 50 °C), effectively inactivated *E. coli* and *A. acidoterrestris* spores, which typically contaminate this type of beverage. However, UV-C/H required less UV fluence than UV-C treatment to obtain similar inactivation. The proposed UV-C/H treatment could represent an alternative to the traditional thermal treatments, which are frequently ineffective in inactivating *A. acidoterrestris* spores in this type of beverage, thus offering a competitive advantage for its industrial application. The sensory analyses performed in this study demonstrated that both UV-C and UV-C/H treated samples were well accepted by consumers, with no significant differences in overall impressions. The ISD, added with a yerba mate extract as a source of caffeine and other bioactive compounds, was well accepted by the consumers, thus representing a niche of opportunities. A group of consumers were identified who displayed high acceptability of this drink with noticeable herbal notes. Moreover, the inclusion of YME enhanced the total phenolic content and antioxidant activity of the drink, offering potential health benefits.

The obtained results could provide a solid basis for producing healthier and more functional sports drinks aligned with current consumption trends and the needs of modern active people. Admittedly, further research is needed to extend the drink’s shelf life and enhance its formulation to meet or exceed consumer expectations.

## Figures and Tables

**Table 1:** Characterization of the ISD systems during refrigerated storage (4 °C, 23 days). (**a**) pH, total soluble solids, turbidity and color parameters and (**b**) total polyphenol content (TPC), total antioxidant activity (TAA) with DPPH or ABTS, flavonoids content (FC) and 5-HMF content.

**(a)**								
**System**	**Storage Time** **(Day)**	**pH**	**TSS** **(°Brix)**	**Turbidity** **(NTU)**	L*	a*	b*	**General ** **Aspect**
ISD-C	0	3.77 ± 0.01 ^a^	6.9 ± 0.1 ^a^	1494 ± 93 ^a^	36.30 ± 0.19 ^a,b^	−2.93 ± 0.44 ^a^	27.94 ± 1.04 ^a^	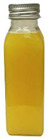
	5	3.84 ± 0.01 ^a^	6.8 ± 0.2 ^a^	1469 ± 426 ^a^	39.88 ± 0.22 ^a^	−4.29 ± 0.17 ^b^	26.36 ± 0.91 ^a,b^	
	9	3.87 ± 0.01 ^a^	6.8 ± 0.1 ^a^	1834 ± 118 ^a^	39.75 ± 0.20 ^a^	−4.29 ± 0.09 ^b^	26.40 ± 0.18 ^a,b^	
	15	3.82 ± 0.01 ^a^	6.4 ± 0.2 ^b^	1574 ± 73 ^a^	38.17 ± 0.09 ^a,b^	−3.80 ± 0.01 ^b^	26.72 ± 0.18 ^a,b^	
	23	3.80 ± 0.01 ^a^	6.6 ± 0.2 ^a,b^	1682 ± 70 ^a^	35.76 ± 0.15 ^b^	−2.83 ± 0.29 ^a^	25.49 ± 0.19 ^b^	
ISD-UVC/H	0	3.76 ± 0.01 ^a^	6.7 ± 0.2 ^a,b^	1584 ± 165 ^a^	36.26 ± 0.18 ^b^	−3.20 ± 0.21 ^a^	26.66 ± 0.32 ^a^	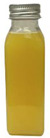
	5	3.84 ± 0.01 ^a^	7.0 ± 0.2 ^a^	1599 ± 142 ^a^	39.72 ± 0.18 ^a^	−4.04 ± 0.07 ^b^	26.07 ± 0.35 ^a^	
	9	3.85 ± 0.02 ^a^	6.4 ± 0.2 ^b,c^	1575 ± 296 ^a^	39.50 ± 0.16 ^a^	−4.18 ± 0.10 ^b^	24.99 ± 1.49 ^a^	
	15	3.80 ± 0.01 ^a^	6.1 ± 0.5 ^a,b,c^	1449 ± 382 ^a^	38.04 ± 0.29 ^a,b^	−3.56 ± 0.12 ^a,b^	26.68 ± 0.48 ^a^	
	23	3.79 ± 0.01 ^a^	6.0 ± 0.1 ^c^	1676 ± 15 ^a^	35.75 ± 0.21 ^a,b^	−2.89 ± 0.14 ^a^	25.04 ± 0.21 ^a^	
ISD-UVC/H+YME	0	3.86 ± 0.01 ^a^	7.1 ± 0.2 ^a^	2151 ± 106 ^a^	34.68 ± 0.22 ^a,b^	−2.52 ± 0.18 ^a^	24.45 ± 0.12 ^a^	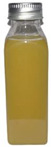
	5	3.97 ± 0.02 ^a^	6.9 ± 0.4 ^a,b^	1665 ± 158 ^b^	38.21 ± 0.12 ^a,b^	−3.52 ± 0.11 ^b^	25.73 ± 0.31 ^a^	
	9	4.00 ± 0.03 ^a^	6.7 ± 0.2 ^a,b^	2116 ± 337 ^a^	38.75 ± 0.26 ^a,b^	−3.42 ± 0.18 ^b^	24.39 ± 1.36 ^a^	
	15	3.88 ± 0.01 ^a^	6.6 ± 0.1 ^b^	2005 ± 30 ^a^	36.88 ± 0.09 ^a,b^	−3.13 ± 0.06 ^b^	25.52 ± 0.84 ^a^	
	23	3.89 ± 0.02 ^a^	6.1 ± 0.4 ^b^	1951 ± 73 ^a,b^	33.73 ± 0.55 ^b^	−2.53 ± 0.77 ^a^	22.64 ± 0.73 ^b^	
**(b)**								
**Sample**	**Time (Day)**	**TPC ** **(mg GAE/mL)**	**TAA_DPPH_** **(mg Trolox Eq/mL)**		**TAA_ABTS_** **(mg Trolox Eq/mL)**	**FC ** **(mg Catechin Eq/mL)**	**HMF ** **(mg HMF/L)**	
ISD-C	0	0.308 ± 0.001 ^a^	0.099 ± 0.025 ^a^		1.348 ± 0.046 ^a^	0.332 ± 0.014 ^a^	0.290 ± 0.096 ^a^	
	5	0.379 ± 0.005 ^a^	0.063 ± 0.023 ^a^		5.026 ± 0.026 ^b^	0.300 ± 0.052 ^a^	0.061 ± 0.069 ^b^	
	9	0.367 ± 0.015 ^a^	0.076 ± 0.012 ^a^		2.710 ± 0.007 ^c^	0.294 ± 0.058 ^a^	0.070 ± 0.001 ^b^	
	15	0.276 ± 0.016 ^a^	0.067 ± 0.073 ^a^		4.170 ± 0.072 ^d^	0.283 ± 0.070 ^a^	0.441 ± 0.011 ^c^	
	23	0.290 ± 0.024 ^a^	0.066 ± 0.075 ^a^		0.834 ± 0.025 ^e^	0.370 ± 0.029 ^a^	0.456 ± 0.096 ^c^	
ISD-UVC/H	0	0.401 ± 0.048 ^a^	0.254 ± 0.077 ^b^		1.208 ± 0.026 ^a^	0.335 ± 0.005 ^a^	0.842 ± 0.169 ^d^	
	5	0.399 ± 0.035 ^a^	0.374 ± 0.007 ^b^		1.730 ± 0.036 ^a^	0.273 ± 0.022 ^a^	0.140 ± 0.107 ^a^	
	9	0.444 ± 0.021 ^a^	0.342 ± 0.015 ^b^		3.959 ± 0.114 ^d^	0.288 ± 0.025 ^a^	0.212 ± 0.051 ^a^	
	15	0.347 ± 0.007 ^a^	0.276 ± 0.020 ^b^		1.453 ± 0.077 ^a^	0.173 ± 0.007 ^a^	0.593 ± 0.051 ^c^	
	23	0.358 ± 0.018 ^a^	0.303 ± 0.041 ^b^		1.292 ± 0.023 ^a,e^	0.361 ± 0.007 ^a^	0.568 ± 0.034 ^c^	
ISD-UVC/H+YME	0	1.149 ± 0.092 ^b^	2.380 ± 0.068 ^d^		8.963 ± 0.016 ^f^	5.541 ± 0.589 ^b^	1.428 ± 0.271 ^e^	
	5	1.420 ± 0.145 ^c^	1.637 ± 0.255 ^e^		9.741 ± 0.051 ^g^	2.197 ± 0.060 ^c^	1.184 ± 0.304 ^d,e^	
	9	1.306 ± 0.349 ^b,c^	1.477 ± 0.035 ^e^		9.119 ± 0.028 ^g^	1.907 ± 0.596 ^c^	1.096 ± 0.316 ^d,e^	
	15	1.080 ± 0.033 ^b^	0.655 ± 0.058 ^f^		6.318 ± 0.028 ^h^	3.756 ± 0.054 ^d^	0.407 ± 0.086 ^c^	
	23	1.416 ± 0.114 ^c^	1.493 ± 0.124 ^e^		7.843 ± 0.016 ^i^	4.434 ± 0.007 ^d^	0.798 ± 0.186 ^d^	

Different lowercase letters (a–i) indicate significant differences (*p* < 0.05) at different storage times, according to the two-way ANOVA with post hoc Tukey’s HSD test. Every value is expressed as mean ± standard deviation. ISD-C: ISD without any treatment; ISD-UVC/H: ISD processed by UV-C light assisted by mild heat (534 mJ/cm^2^, 50 ± 1 °C); ISD-UVC/H+YME: processed ISD loaded with YME.

## Data Availability

The original contributions presented in the study are included in the article, further inquiries can be directed to the corresponding author.
